# The Rsm regulon of plant growth-promoting *Pseudomonas fluorescens* SS101: role of small RNAs in regulation of lipopeptide biosynthesis

**DOI:** 10.1111/1751-7915.12190

**Published:** 2014-12-09

**Authors:** Chunxu Song, Menno van der Voort, Judith van de Mortel, Karl A Hassan, Liam D H Elbourne, Ian T Paulsen, Joyce E Loper, Jos M Raaijmakers

**Affiliations:** 1 Laboratory of Phytopathology, Wageningen University6708 PD, Wageningen, the Netherlands; 2 Department of Chemistry and Biomolecular Sciences, Macquarie University2109, Sydney, NSW, Australia; 3 Horticultural Crops Research Laboratory, USDA-ARSCorvallis, OR, USA; 4 Department of Microbial Ecology, Netherlands Institute of Ecology6708 PB, Wageningen, the Netherlands

## Abstract

The rhizobacterium *Pseudomonas fluorescens* SS101 inhibits growth of oomycete and fungal pathogens, and induces resistance in plants against pathogens and insects. To unravel regulatory pathways of secondary metabolite production in SS101, we conducted a genome-wide search for sRNAs and performed transcriptomic analyses to identify genes associated with the Rsm (repressor of secondary metabolites) regulon. *In silico* analysis led to the identification of 16 putative sRNAs in the SS101 genome. In frame deletion of the sRNAs *rsmY* and *rsmZ* showed that the Rsm system regulates the biosynthesis of the lipopeptide massetolide A and involves the two repressor proteins RsmA and RsmE, with the LuxR-type transcriptional regulator MassAR as their most likely target. Transcriptome analyses of the *rsmYZ* mutant further revealed that genes associated with iron acquisition, motility and chemotaxis were significantly upregulated, whereas genes of the type VI secretion system were downregulated. Comparative transcriptomic analyses showed that most, but not all, of the genes controlled by RsmY/RsmZ are also controlled by the GacS/GacA two-component system. We conclude that the Rsm regulon of *P. fluorescens* SS101 plays a critical role in the regulation of lipopeptide biosynthesis and controls the expression of other genes involved in motility, competition and survival in the plant rhizosphere.

## Introduction

Computational searches of intergenic regions, promoters and rho-independent transcription terminators (Livny *et al*., 2005; 2006; Sridhar and Gunasekaran, 2013; Wright *et al*., 2013) combined with experimental approaches (Sharma and Vogel, 2009) have revealed the presence of several small RNAs (sRNAs) in bacterial genomes. In general, two types of regulatory sRNAs have been described (Majdalani *et al*., 2005; Gottesman *et al*., 2006; Pichon and Felden, 2007; Gottesman and Storz, 2011). The first targets specific messenger RNAs (mRNAs) by base pairing. An example is RyhB in *Escherichia coli* which interacts with the mRNA encoding SodB, an iron-containing superoxide dismutase (Salvail *et al*., 2010). The second type interacts with RNA-binding proteins of the RsmA/CsrA family. RsmA (regulator of secondary metabolism) and CsrA (carbon storage regulator) act as translational repressors and their sequestration by activated sRNAs can relieve repression of the target mRNAs.

In *Pseudomonas*, relatively few sRNAs have been studied in detail for their functions. In *Pseudomonas protegens* strain CHA0, the sRNAs RsmX, RsmY and RsmZ are under the control of the GacS/GacA two-component system and regulate the production of a range of secondary metabolites (Heeb *et al*., 2002a; Valverde *et al*., 2003; Kay *et al*., 2005; Lapouge *et al*., 2007; 2008). In *P. protegens* CHA0, Gac/Rsm-mediated regulation of secondary metabolites involves sequestration of the repressor proteins RsmA and RsmE that act post-transcriptionally by binding to the target mRNA (Blumer *et al*., 1999; Reimmann *et al*., 2005; Lapouge *et al*., 2008). In *Pseudomonas aeruginosa*, the two sRNAs, RsmY and RsmZ, regulate quorum sensing and the biosynthesis of several exoproducts (Brencic *et al*., 2009; Frangipani *et al*., 2014). Other sRNAs described for *P. aeruginosa* are PhrS, PrrF1 and PrrF2: PhrS is involved in the regulation of quinolone biosynthesis (Sonnleitner and Haas, 2011; Sonnleitner *et al*., 2011), and PrrF1 and PrrF2 contribute to iron acquisition (Wilderman *et al*., 2004; Sonnleitner and Haas, 2011).

Most of the known sRNAs in *Pseudomonas* and other Gram-negative bacterial genera are under the control of the Gac/Rsm signal transduction pathway. Based on the proposed model, the phosphorylated regulator GacA binds to a conserved element upstream of the sRNA promoter, referred to as the GacA box, to activate their expression (Lapouge *et al*., 2008). In many cases, mutations or deletions of the sRNAs result in phenotypes similar to that of GacS/GacA mutants. For example, Δ*rsmYZ* and Δ*gacA* mutants of *P. aeruginosa* are both deficient in the synthesis of the quorum sensing signal N-butanoyl-homoserine lactone, hydrogen cyanide (HCN), pyocyanin, elastase and chitinase as well as in biofilm formation (Kay *et al*., 2006; Brencic *et al*., 2009). In *Pseudomonas entomophila*, Δ*rsmYZ* and Δ*gacA* mutants were both deficient in the production of entolysin (Vallet-Gely *et al*., 2010). Similarities in phenotypes of *rsm* and *gac* mutants have also been described for *Pectobacterium carotovorum* (Liu *et al*., 1998), *E. coli* (Weilbacher *et al*., 2003), *Salmonella enterica* (Fortune *et al*., 2006) and *Legionella pneumophila* (Sahr *et al*., 2009).

In this study, we conducted a genome-wide search for sRNAs in *Pseudomonas fluorescens* strain SS101 and performed transcriptomic analyses to identify genes associated with the Rsm regulon and with the Gac regulon. We addressed the function of the Rsm regulon, involving the two sRNAs RsmY (PflSS101_4962) and RsmZ (PflSS101_1168), and the two repressor proteins RsmA (PflSS101_4138) and RsmE (PflSS101_3491), in lipopeptide biosynthesis and predicted the potential target genes of the Rsm repressor proteins. Strain SS101 was originally isolated from the rhizosphere of wheat (de Souza *et al*., 2003), has activity against various oomycete and fungal pathogens (de Souza *et al*., 2003; Tran *et al*., 2013; van de Mortel *et al*., 2009) and induces systemic resistance in tomato and Arabidopsis against several pathogens and insect pests (Tran *et al*., 2013; van de Mortel *et al*., 2012). Comparative genome analyses of multiple *Pseudomonas* species and strains (Loper *et al*., 2012) revealed that strain SS101 harbours 350 unique genes, which include prophage and genomic islands. Unlike many other *P. fluorescens* and *P. protegens* biocontrol strains, SS101 does not produce the typical secondary metabolites such as 2,4-diacetylphloroglucinol (DAPG), phenazines, pyrrolnitrin, pyoluteorin and HCN (Loper *et al*., 2012). The main secondary metabolite produced by SS101 is the cyclic lipopeptide massetolide A, whose biosynthesis is governed by the non-ribosomal peptide synthetase (NRPS) genes *massABC* and regulated by the GacS/GacA system (de Bruijn and Raaijmakers, 2009a). Massetolide A contributes to biofilm formation, swarming motility, antimicrobial activity and defense against protozoan predators (Mazzola *et al*., 2009; Raaijmakers *et al*., 2010). Here, genome-wide transcriptional analysis of mutants with deletions in *rsmY* and *rsmZ* revealed that the NRPS genes *massA*, *massB*, *massC* as well as the LuxR-type transcriptional regulator *massAR* were significantly downregulated. Via mutational and phenotypic analyses, we show that the Rsm system regulates massetolide biosynthesis as well as several other genes and traits in the rhizobacterium *P. fluorescens* SS101.

## Results and discussion

### Small RNAs in *P. fluorescens* SS101

A total of 68 tRNAs and 19 rRNAs were found in the SS101 genome (Table S1). Genome-wide analyses revealed 16 predicted sRNAs including homologues of the two signal recognition particle RNAs SrpB_1 (PflSS101_3911) and SrpB_2 (PflSS101_3926) (Table 1). Signal recognition particle (Srp) is a ribonucleoprotein complex that participates in multiple protein targeting pathways in bacteria (Koch *et al*., 1999) and is primarily involved in the incorporation of proteins in the inner membrane (Rosenblad *et al*., 2009). Furthermore, we also found a 6S SsrS RNA (PflSS101_5226) in the SS101 genome. In *E. coli*, 6S RNA is encoded by the *ssrS* gene which regulates transcription during late exponential and stationary growth (Wassarman, 2007). Bacterial Ribonuclease P (PflSS101_0956) was found in the SS101 genome and represents a ribonucleoprotein complex comprised of a single RNA (∼ 400 nt) and a single small protein subunit (∼ 14 kDa) with the RNA as the catalytic subunit of the enzyme involved in the maturation of tRNA transcripts (Ellis and Brown, 2009). We also found homologues of PhrS (PflSS101_4081), PrrF1 (PflSS101_4589) and PrrF2 (PflSS101_3274), which are known to repress or activate the translation of target mRNAs by a base pairing mechanism. In *P. aeruginosa*, the two *prrF* sRNA genes are found in tandem. Homologous genes in other *Pseudomonas* species are located considerably distant from each other on the chromosome (Wilderman *et al*., 2004). Also in SS101, PrrF1 (PflSS101_4589) and PrrF2 (PflSS101_3274) are found at different locations in the genome. We also found RgsA (PflSS101_1357) in the SS101 genome, which is an sRNA probably regulated indirectly by GacA and directly by the stress sigma factor RpoS (Gonzalez *et al*., 2008).

**Table 1:**
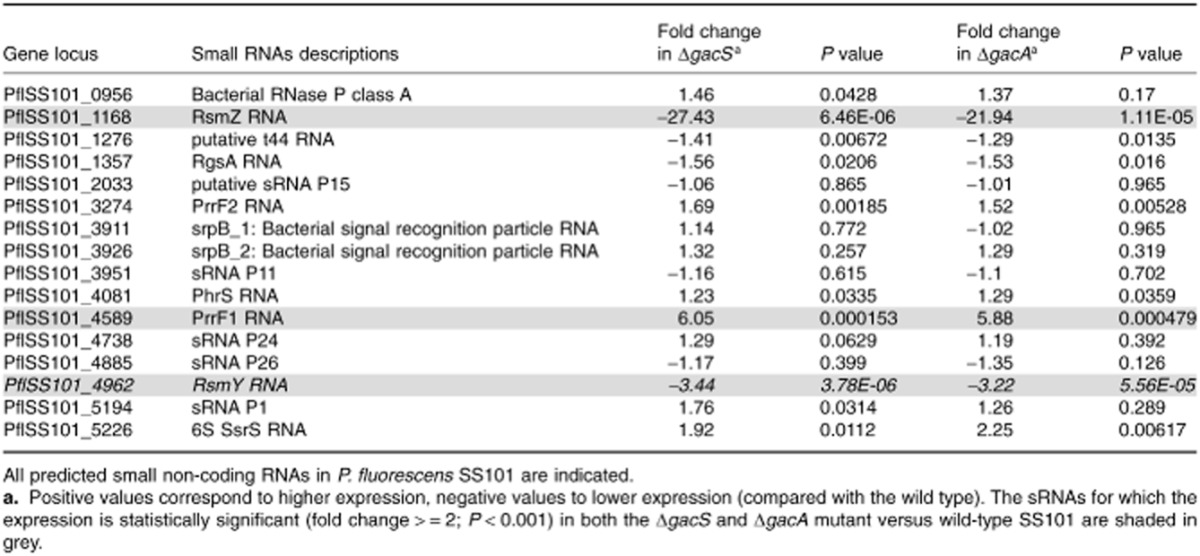
Small non-coding RNAs in *P. fluorescens* SS101

Two other sRNAs found in the SS101 genome were RsmY (PflSS101_4962) and RsmZ (PflSS101_1168) (Table 1). In *P. protegens* and *P. aeruginosa*, RsmY and RsmZ regulate secondary metabolite production by sequestering RNA-binding proteins (e.g. CsrA, RsmA) that act as translational repressors (Kay *et al*., 2005; Gottesman and Storz, 2011). In *P. aeruginosa*, the expression of all Gac-regulated genes was shown to be RsmY/Z dependent (Brencic *et al*., 2009). For the other sRNAs detected in the SS101 genome (Table 1), the functions are poorly understood or not known from other *Pseudomonas* species. Here, we will specifically focus on the sRNAs in strain SS101 that are regulated by the GacS/GacA two-component system.

### Small RNAs in *P. fluorescens* SS101 regulated by the GacS/A system

**Figure 1:**
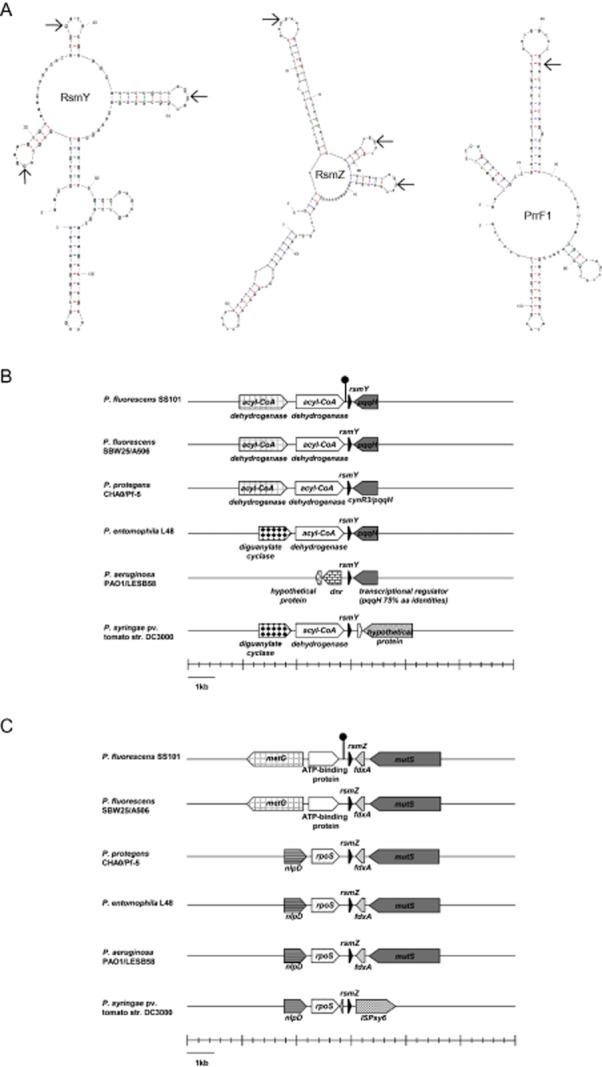
Secondary structures of small RNAs, RsmY, RsmZ, PrrF1 in *P. fluorescens* SS101 and the genetic organization of *rsmY* and *rsmZ* in strain SS101 and other *Pseudomonas* species and strains.A. Predicted secondary structures of RsmY, RsmZ and PrrF1 of *P. fluorescens* SS101 by MFOLD (http://mfold.rna.albany.edu/?q=mfold/RNA-Folding-Form). The typical GGA motifs located in the loop regions are indicated with arrows.B. Genetic organization of *rsmY* regions in different *Pseudomonas* species and strains. Block arrows indicate directionality of the open reading frame, and orthologous genes are represented by color and pattern. The loop symbol in front of *rsmY* indicates the position of the upstream activating sequence (UAS for *rsmY*: TGTAAGCATTCTCTTACA). Abbreviations: *pqqH*/*cynR3/dnr*: transcriptional regulator.C. Genetic organization of *rsmZ* regions in different *Pseudomonas* species and strains. Block arrows indicate directionality of the open reading frame, and orthologous genes are represented by colour and pattern. The loop symbol in front of *rsmZ* indicates the position of the UAS (UAS for *rsmZ*: TGTAAGCATTCGCTTACT). Abbreviations: *metG*: methionyl-tRNA synthetase; *fdxA*: ferredoxin; *mutS*: DNA mismatch repair protein; *nlpD*: lipoprotein; *rpoS*: RNA polymerase sigma factor; ISPsy6: transposase.

Transcriptomic analyses of both *gacS* and *gacA* mutants of *P. fluorescens* SS101 (Tables S2, S3) revealed that the expression of three sRNAs (*rsmY, rsmZ* and *prrF1*) was significantly (> 2-fold, *P* < 0.001) altered (Table 1). Expression of *rsmY* and *rsmZ* was significantly downregulated in both *gacS* and *gacA* mutants, whereas expression of *prrF1* was approximately six-fold upregulated in both *gac* mutants. The predicted sizes of the *rsmY, rsmZ* and *prrF1* transcripts were 118 bp, 133 bp and 112 bp respectively. Subsequent prediction of their secondary structures revealed eight GGA motifs in both RsmY and RsmZ, with three in predicted loop regions respectively (Fig. 1A). In contrast, only one GGA motif was found in PrrF1, which is localized to a predicted stem (Fig. 1A). Repeated GGA motifs in loop regions of the secondary structure, as predicted for RsmY and RsmZ, are an essential characteristic of sRNAs for sequestration of RsmA and homologous repressor proteins (Lapouge *et al*., 2008). Previous work also showed that the regions upstream of these sRNAs contain a conserved 18 bp sequence which corresponds to the GacA-binding site for activation of these sRNAs (Heeb *et al*., 2002b; Kay *et al*., 2005). For SS101, we indeed found this typical GacA-binding box upstream of *rsmY* and *rsmZ* (Fig. 1B and C), but not for *prrF1*. Therefore, our subsequent functional analyses focused on *rsmY* and *rsmZ*.

### Role of RsmY and RsmZ in lipopeptide biosynthesis in *P. fluorescens* SS101

**Figure 2:**
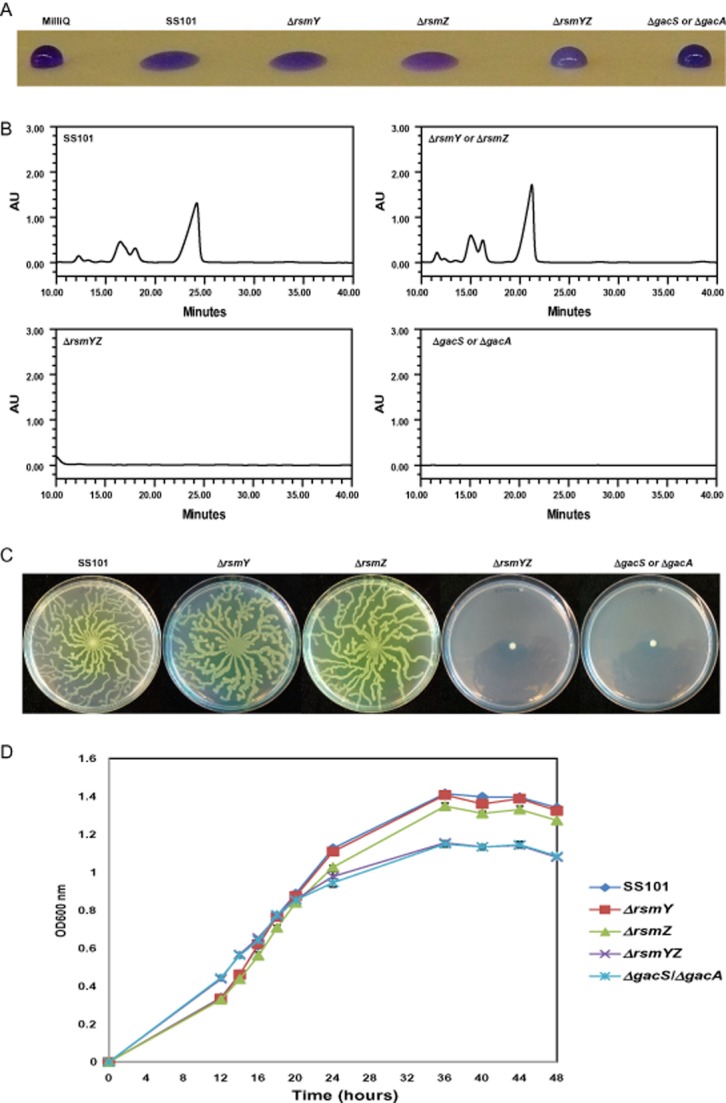
Phenotypic and chemical analyses of *P. fluorescens* strain SS101 and single or double mutants disrupted in *rsmY*, *rsmZ*, *gacS* or *gacA*.A. Drop collapse assay with cell cultures of wild-type strain SS101, Δ*rsmY*, Δ*rsmZ*, Δ*rsmYZ*, Δ*gacS* and Δ*gacA* mutants. Bacterial cultures grown for 2 days at 25°C on KB agar plates were suspended in sterile water to a final density of 1 × 10^10^ cells ml^−1^, and 10-μl droplets were spotted on parafilm, and crystal violet was added to the droplets to facilitate visual assessment. A flat droplet is a highly reliable proxy for the production of the surface-active lipopeptide massetolide A.B. Reversed phase-high-performance liquid chromatography chromatograms of cell-free culture extracts of wild-type strain SS101, Δ*rsmY*, Δ*rsmZ*, Δ*rsmYZ*, Δ*gacS* and Δ*gacA* mutants as described in A. The wild-type strain SS101 produces massetolide A (retention time of approximately 23–25 min) and various other derivatives of massetolide A (minor peaks with retention times ranging from 12 to 18 min) which differ from massetolide A in the amino acid composition of the peptide moiety. AU stands for absorbance unit.C. Swarming motility of wild-type strain SS101, Δ*rsmY*, Δ*rsmZ*, Δ*rsmYZ*, Δ*gacS* and Δ*gacA* mutants on soft [0.6% (wt/vol)] agar plates. Five microlitres (1 × 10^10^ cells ml^−1^) of washed overnight cultures of wild-type SS101 or mutants were spot inoculated in the centre of a soft agar plate and incubated for 48 to 72 h at 25°C.D. Growth of wild-type strain SS101, Δ*rsmY*, Δ*rsmZ*, Δ*rsmYZ*, Δ*gacS* and Δ*gacA* mutants in liquid broth at 25°C. At different time points, the optical density of the cell cultures was measured spectrophotometrically (600 nm). Mean values of four biological replicates are given; the error bars represent the standard error of the mean.

The location of *rsmY* and *rsmZ* in the genomes appears to be conserved, at least to some extent, for the different *Pseudomonas* species and strains (Fig. 1B and C). In frame deletion, mutants were generated to investigate the role of *rsmY* and *rsmZ* in the regulation of massetolide A biosynthesis. The drop collapse assay, a reliable proxy for detection of massetolide A and other lipopeptide surfactants (de Bruijn *et al*., 2008; de Bruijn and Raaijmakers, 2009a), showed that mutations in either *rsmY* or *rsmZ* alone did not affect massetolide A production (Fig. 2A). However, mutations in both *rsmY* and *rsmZ* resulted in loss of massetolide A production which was confirmed by reversed phase-high-performance liquid chromatography (RP-HPLC) (Fig. 2B). Also swarming motility of SS101, a phenotype that depends on massetolide production (de Bruijn *et al*., 2008), was abolished in the *rsmYZ* double mutant (Fig. 2C). Mutations in *rsmY* or *rsmZ* alone did not affect growth of strain SS101 (Fig. 2D). However, mutations in both *rsmY* and *rsmZ* slightly enhanced growth in the early exponential phase but had an adverse effect on growth during the late exponential and stationary phase; similar changes in growth dynamics were observed for the *gacS* and *gacA* mutants of strain SS101 (Fig. 2D). These changes in growth dynamics are most likely not related to a lack of massetolide production, because growth of the site-directed *massA* biosynthesis mutant of SS101 was similar to that of the wild type (de Bruijn and Raaijmakers, 2009a). In summary, these results indicated that both RsmY and RsmZ are an integral component of the GacS/GacA signal transduction cascade and regulate massetolide biosynthesis in *P. fluorescens* SS101.

### Deletion of repressor proteins restores massetolide production

**Figure 3:**
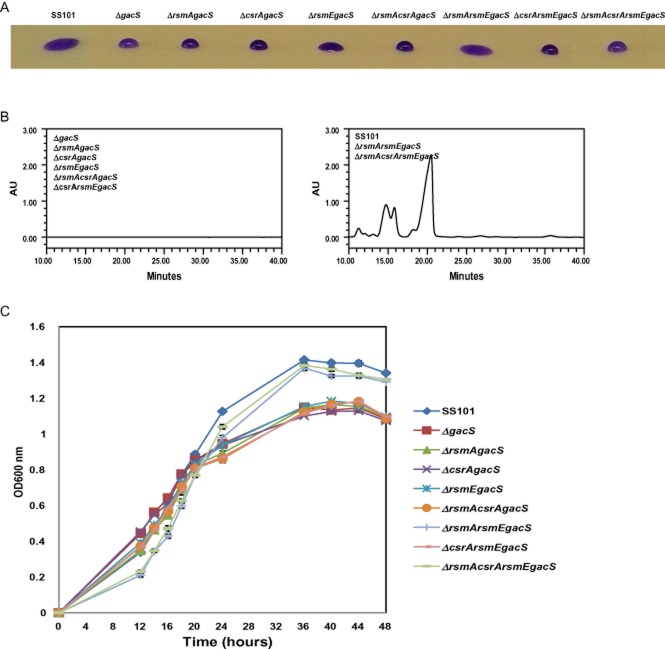
Phenotypic and chemical analyses of *P. fluorescens* strain SS101, Δ*gacS* mutant and single, double or triple mutants disrupted in *rsmA*, *rsmE* and *csrA* in the Δ*gacS* background.A. Drop collapse assay with cell suspensions of wild-type SS101, Δ*gacS*, Δ*rsmAgacS*, Δ*csrAgacS*, Δ*rsmEgacS*, Δ*rsmAcsrAgacS*, Δ*rsmArsmEgacS*, Δ*csrArsmEgacS* and Δ*rsmAcsrArsmEgacS* mutants. Bacterial cultures grown for 2 days at 25°C on KB agar plates were suspended in sterile water to a final density of 1 × 10^10^ cells ml^−1^, and 10-μl droplets were spotted on parafilm, and crystal violet was added to the droplets to facilitate visual assessment. A flat droplet is a highly reliable proxy for the production of the surface-active lipopeptide massetolide A.B. Reversed phase-high-performance liquid chromatography chromatograms of cell-free culture extracts of wild-type SS101, Δ*rsmAgacS*, Δ*csrAgacS*, Δ*rsmEgacS*, Δ*rsmAcsrAgacS*, Δ*rsmArsmEgacS*, Δ*csrArsmEgacS* and Δ*rsmAcsrArsmEgacS* mutants as described in A. The wild-type strain SS101 produces massetolide A (retention time of approximately 18–21 min) and various other derivatives of massetolide A (minor peaks with retention times ranging from 12 to 18 min) which differ from massetolide A in the amino acid composition of the peptide moiety. AU stands for absorbance unit. Representative chromatograms of Δ*rsmAgacS* and Δ*rsmArsmEgacS* mutants are shown.C. Growth of wild-type SS101, Δ*rsmAgacS*, Δ*csrAgacS*, Δ*rsmEgacS*, Δ*rsmAcsrAgacS*, Δ*rsmArsmEgacS*, Δ*csrArsmEgacS* and Δ*rsmAcsrArsmEgacS* mutants in liquid broth at 25°C. At different time points, the optical density of the cell cultures was measured spectrophotometrically (600 nm). Mean values for four biological replicates are given; the error bars represent the standard errors of the mean.

Previous studies with *P. protegens* CHA0 have shown that Gac/Rsm-mediated regulation of secondary metabolites involves sequestration of the repressor proteins RsmA and RsmE that act post-transcriptionally by binding to the target mRNA (Blumer *et al*., 1999; Reimmann *et al*., 2005; Lapouge *et al*., 2008). Hence, the next step was to determine if these repressor proteins are present in SS101 and if they play a role in Gac/Rsm-mediated regulation of massetolide biosynthesis. *In silico* analysis of the SS101 genome led to the identification of *rsmA* (PflSS101_4138), *rsmE* (PflSS101_3491) and *csrA* (PflSS101_3653). Phylogenetic analyses showed that they clustered closely with their homologues in other *P. fluorescens* strains and *Pseudomonas* species at both DNA and protein levels (Fig. S1). To decipher their role in regulation of massetolide biosynthesis, deletion mutants were made for each of these three repressors in the *gacS* mutant background of strain SS101. The *gacS* mutant does not produce massetolide, but according to the regulatory model, a mutation of the repressor proteins would alleviate translational repression and restore production. The results of the drop collapse assay and RP-HPLC analyses showed that a deletion of either *rsmA* or *csrA* in the *gacS* mutant did not restore massetolide production (Fig. 3A and B). Based on the drop collapse assay, a mutation in the *rsmE* gene partially affected the surface tension (Fig. 3A), but massetolide production was not detectable by RP-HPLC analysis (Fig. 3B). A double mutation in *rsmE* and *rsmA* fully restored massetolide production (Fig. 3A and B). A single deletion of either one of the repressor genes did not affect growth as compared with that of the *gacS* mutant, whereas stacked deletions of *rsmA* and *rsmE* in the *gacS* mutant changed the growth dynamics back to that of the wild type (Fig. 3C). We conclude that Gac/Rsm-mediated regulation of massetolide biosynthesis via *rsmY* and *rsmZ* implicates the two small RNA binding proteins RsmA and RsmE, whereas CsrA is not involved.

### Potential targets of the RsmA/RsmE repressor proteins in *P. fluorescens* SS101

**Figure 4:**
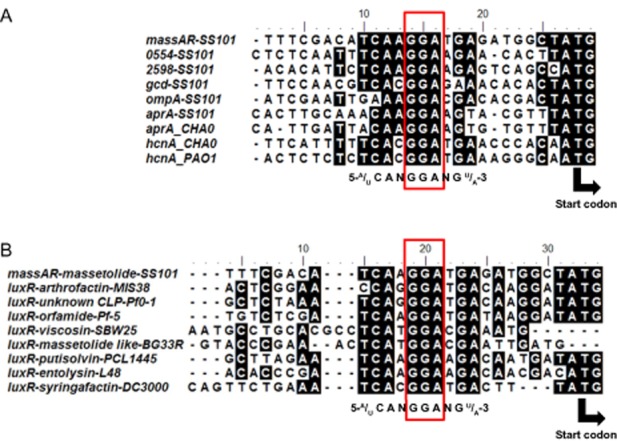
A. Alignment of the upstream regions of five putative target genes of the RsmA and RsmE repressor proteins of *P. fluorescens* SS101. The *aprA* and *hcnA* genes of *P. protegens* CHA0 and *P. aeruginosa* PAO1 were used as references. The translation initiation ATG codon is shown at the 3′ end. B. Alignment of the regions upstream of LuxR-type transcriptional regulatory genes that flank different lipopeptide biosynthesis gene clusters in *Pseudomonas fluorescens* SS101, *Pseudomonas* sp. MIS38, *P. fluorescens* Pf0-1, *P. protegens* Pf-5, *P. fluorescens* SBW25, *P. synxantha* BG33R, *P. putida* PCL1445, *P. entomophila* L48 and *Pseudomonas syringae* pv. *tomato* DC3000. The translation initiation ATG codon is shown at the 3′ end.

To determine the potential targets of the RsmA and RsmE repressor proteins, we conducted a whole genome search for putative Rsm binding sites at or near the 5′ untranslated leader mRNA by using the conserved motif 5′-^A^/_U_ CANGGANG^U^/_A_-3′ (N is any nucleotide) (Lapouge *et al*., 2008). A total of 17 genes were found with this conserved motif located in the ribosome binding site (RBS) (Table 2). For six of these 17 genes, transcription was significantly downregulated in the *gacS*/*gacA* mutants and also in the *rsmYZ* double mutant (Table 2). These six genes included: PflSS101_0554 with unknown function; *gcd* (PflSS101_1096) encoding the quinoprotein glucose dehydrogenase; *ompA* (PflSS101_1239); *aprA* (PflSS101_2560), which encodes an extracellular protease; PflSS101_2598, a gene predicted to encode a formyl-transferase domain/enoyl-CoA hydratase/isomerase family protein; and *massAR* (PflSS101_3396), the LuxR-type transcriptional regulatory gene located upstream of the *massA* biosynthesis gene and essential for massetolide biosynthesis (de Bruijn and Raaijmakers, 2009a,b). There was no GacA box sequence upstream of *massA*, *massBC* or *massBC*R (LuxR type regulator downstream of *massBC*). Alignment of the 5′ untranslated leader regions of these six putative target genes, with *hcnA* and *aprA* of *P. protegens* CHA0 and *P. aeruginosa* PAO1 as references, revealed the position of the consensus motif close to the RBS (Fig. 4A). When the alignment for *massAR* was performed with genes of several closely related LuxR-type transcriptional regulator genes flanking other lipopeptide biosynthesis genes in different *Pseudomonas* species and strains, similar consensus motifs were found (Fig. 4B). Based on these findings, we postulate that (i) the LuxR-type transcriptional regulator MassAR is the most likely target of the RsmA and RsmE repressor proteins in Gac/Rsm-mediated regulation of massetolide biosynthesis in *P. fluorescens* SS101; and (ii) lipopeptide biosynthesis in other *Pseudomonas* species is most likely regulated in a similar manner.

**Table 2:**
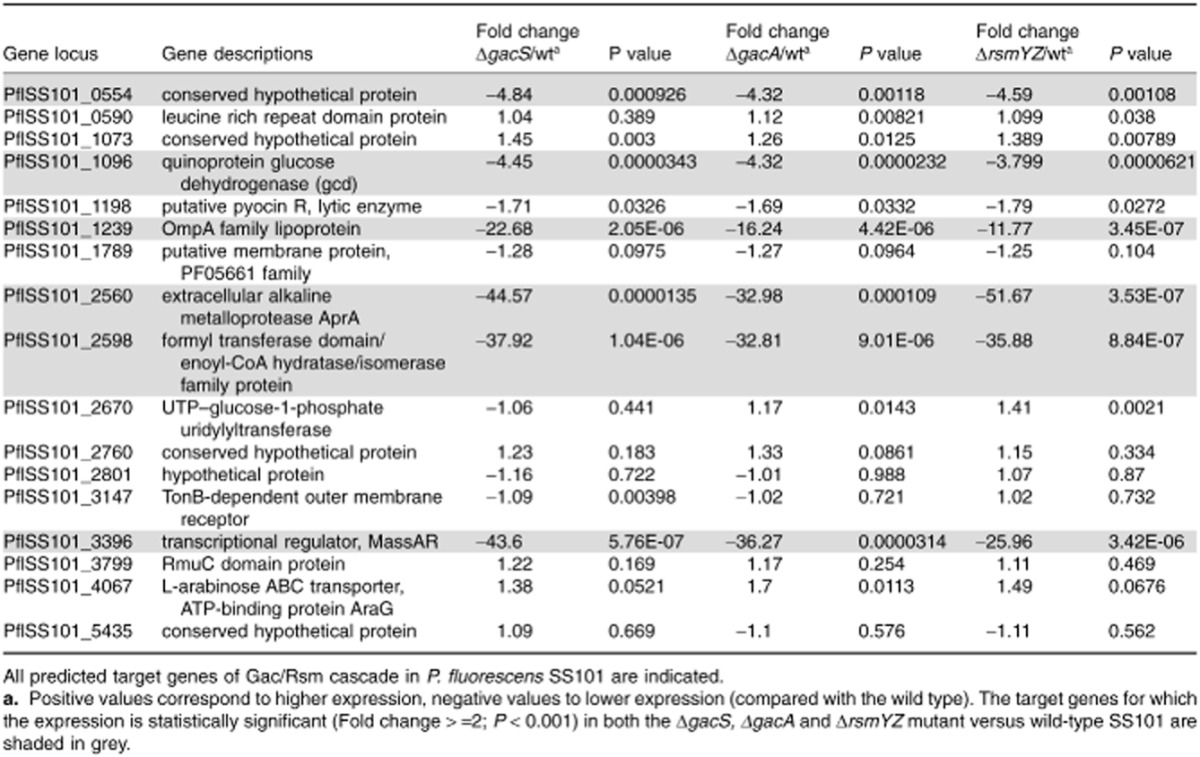
Predicted target genes of the RsmA and RsmE repressor proteins in *P. fluorescens* SS101

### Other genes of the Rsm regulon in *P. fluorescens* SS101

**Figure 5:**
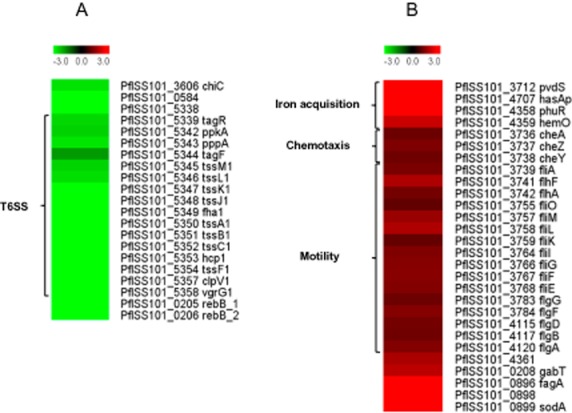
Whole genome transcriptome analysis of *P. fluorescens* SS101 and the Δ*rsmYZ* mutant. Heat maps showing significant log_2_-fold changes (*P* < 0.001) in the expression of genes in the Δ*rsmYZ* versus wild-type cells. Wild-type SS101 and the Δ*rsmYZ* mutant were grown in liquid KB at 25°C to an optical cell density of OD_600_ = 0.6. The fold changes shown here represent averages of three biological replicates. A represents known genes that were downregulated in the Δ*rsmYZ* mutant, whereas B represents known genes upregulated in the Δ*rsmYZ* mutant versus wild-type SS101. For a list of all genes differentially regulated in the Δ*rsmYZ* mutant versus wild-type SS101, we refer to Tables S2 and S3.

To explore the potential roles of *rsmY* and *rsmZ* in global gene regulation in strain SS101, we conducted a genome-wide microarray analysis on the *rsmYZ* double mutant and the wild-type strain, both sampled in the mid-exponential growth phase (OD_600_ ∼ 0.6). In *rsmYZ*, the expression of *rsmY* and *rsmZ* was reduced 89 and 82-fold, respectively, due to the deletion of the corresponding genes. Various other significant changes in gene expression were observed with 121 and 272 genes significantly (fold change > 2.0; *P* < 0.001) up- and downregulated respectively (Table S4; Table S5). Next to the genes involved in massetolide biosynthesis, the chitinase encoding gene *chiC* (PflSS101_3606) and a gene predicted to encode a bacterioferritin family protein (PflSS101_0584) were significantly downregulated in the *rsmYZ* mutant. Moreover, 19 genes (PflSS101_5338–5358) homologous to the HSI-I type VI secretion system of *P. aeruginosa* (Mougous *et al*., 2006) were downregulated (Fig. 5A). Another type VI secretion system HSI-II was not differentially regulated in the *rsmYZ* mutant. The putative functions of these type VI secretion systems in SS101, including a role in antibacterial activity or in plant-growth promotion (Decoin *et al*., 2014), are yet unknown.

Transcriptomic analysis also revealed that *rebB_1* (PflSS101_0205) and *rebB_2* (PflSS101_0206) were downregulated more than 44-fold and 93-fold, respectively, in the *rsmYZ* mutant (Table S3). For certain endosymbionts, such as *Caedibacter* in *Paramecium*, these genes have been reported to encode insoluble proteins referred to as refractile bodies (R bodies) (Schrallhammer *et al*., 2012). It has been noted that R bodies unwind under certain conditions and are associated with toxicity, i.e. the ability to kill symbiont-free competitors. For free-living bacteria, including *P. fluorescens* SS101, the functions of these R bodies are not known yet. Given that not all downregulated genes in *rsmYZ* double mutant harbour the conserved motif 5′-^A^/_U_ CANGGANG^U^/_A_-3′ in the ribosome-binding site (data not shown), we postulate that the altered expression of these genes might be due to indirect regulation by the Rsm regulon as was reported for *P. aeruginosa* (Brencic and Lory, 2009).

Genes upregulated in the *rsmYZ* mutant represent genes involved in iron acquisition, chemotaxis and cell motility (Fig. 5B). Also, *gabT* (PflSS101_0208), which is involved in γ-aminobutyric acid utilization, was upregulated in the *rsmYZ* mutant. Upregulation was also found for three genes of the *fagA-fumC-orfX-sodA* operon (PflSS101_0896, 0898, 0899) (Fig. 5B), which functions in oxidative stress adaptation in *P. aeruginosa* (Polack *et al*., 1996; Hassett *et al*., 1997a,b).

### Comparison of the Rsm regulon and the Gac regulon of *P. fluorescens* SS101

Many of the genes differentially regulated in the *rsmYZ* mutant of strain SS101 have also been reported previously to be differentially expressed in Gac mutants of other *Pseudomonas* species and strains (Brencic *et al*., 2009; Hassan *et al*., 2010; Cheng *et al*., 2013; Wang *et al*., 2013). In *P. aeruginosa*, the GacS/GacA transduction system acts exclusively through its control over the transcription of *rsmY* and *rsmZ* (Brencic *et al*., 2009). However, the possibility that the system directly regulates other genes cannot be excluded for other *Pseudomonas* species and strains. For instance, in *L. pneumophila*, LetA (orthologue of GacS) regulates expression of flagellar genes by a mechanism that appears to be independent of RsmY and RsmZ (Sahr *et al*., 2009). In our study, comparative analyses of the Gac regulon and Rsm regulon of *P. fluorescens* SS101 were conducted according to Sahr and colleagues (2009). Briefly, we made a direct comparison (fold change > 2.0, *P* value < 0.05) of the gene expression pattern of Δ*gacA* and Δ*rsmYZ*. Additionally, we analysed genes differentially expressed in either Δ*gacA/wt* or in Δ*rsmYZ/wt*. Collectively, these analyses resulted in five genes differentially expressed in the Δ*gacA* mutant and 11 genes differentially expressed in the Δ*rsmYZ* mutant. One of the five genes (PflSS101_2039) that was differentially expressed in the Δ*gacA* mutant is located directly downstream of *gacA*. Hence, its differential expression is most likely due to a polar effect of the *gac* mutation. Therefore, this gene was excluded from the comparison. In summary, the expression of four and 11 genes varied in Δ*gacA* and Δ*rsmYZ* mutants respectively. One of these four genes is related to iron uptake, one is involved in amino acid transport and metabolism, and two genes are predicted to encode a hypothetical protein. The 11 genes uniquely expressed in the *rsmYZ* mutant (Table S6) were all significantly upregulated. One gene, encoding a secondary thiamine-phosphate synthase enzyme, showed the most increased expression (nine-fold change), but its function in strain SS101 is not known yet. In summary, this analysis suggests that most, not all, of the genes controlled by GacS/GacA two-component system are controlled via RsmY/RsmZ.

## Conclusions

Through *in silico* analyses of the genome of the rhizobacterium *P. fluorescens* SS101, 16 small RNAs were identified. Subsequent experiments revealed, for the first time, that the Rsm signal transduction pathway plays a critical role in the regulation of massetolide biosynthesis, a cyclic lipopeptide important for biofilm formation, swarming motility, antimicrobial activity and induction of systemic resistance in plants. We showed that the effects of the two sRNAs RsmY and RsmZ are channeled through the RsmA and RsmE repressor proteins, and we predicted that the LuxR-type transcriptional regulator MassAR is one of the targets of these repressor proteins in strain SS101. To date, most information on the Rsm regulon in *Pseudomonas* species comes from studies on *P. aeruginosa* and *P. protegens*. Here, new information is provided that the Rsm system regulates lipopeptide biosynthesis in *P. fluorescens* SS101 and possibly other *Pseudomonas* species. Our study also provided, for the first time, a whole genome comparison of the Rsm and Gac regulons in a *Pseudomonas* species other than *P. aeruginosa*. The results of these analyses revealed that most but not all of the genes controlled by RsmY/RsmZ are also controlled by the GacS/GacA two-component system, whereas in *P. aeruginosa*, the Gac regulon controls downstream genes exclusively through the sRNAs RsmY and RsmZ.

## Experimental procedures

### Bioinformatic prediction of sRNAs in *P. fluorescens* SS101 genome

sRNA searches were performed by blast and yass (Noe and Kucherov, 2005) against the Rfam database (http://rfam.janelia.org/), as well as by erpin (Gautheret and Lambert, 2001), infernal (Nawrocki *et al*., 2009) and darn (Zytnicki *et al*., 2008), which are included in the rnaspace package (Cros *et al*., 2011).

### Bacterial strains and cultural conditions

Bacterial strains used in this study are listed in Table 3. *Pseudomonas fluorescens* strains were cultured in liquid King's medium B (KB) (King *et al*., 1954) at 25°C. The *gacS* and *gacA* plasposon mutants were obtained with plasmid pTnModOKm (Dennis and Zylstra, 1998). *Escherichia coli* strain DH5α was used as a host for the plasmids used for site-directed mutagenesis. *Escherichia coli* strains were grown on Luria–Bertani (LB) plates or in LB broth (Bertani, 1951) amended with the appropriate antibiotics.

**Table 3:** Bacterial strains and mutants used in this study

Strain	Relative characteristics	Reference source
*Pseudomonas fluorescens*		
SS101	Wild type, Rif^r^	de Souza *et al*., 2003
Δ*gacS*	Plasposon mutant, Km^r^	This study
Δ*gacA*	Plasposon mutant, Km^r^	This study
Δ*rsmY*	*rsmY* deletion mutant	This study
Δ*rsmZ*	*rsmZ* deletion mutant	This study
Δ*rsmYZ*	*rsmY rsmZ* deletion mutant	This study
Δ*rsmAgacS*	*rsmA* deletion mutant in the Δ*gacS* background	This study
Δ*csrAgacS*	*csrA* deletion mutant in the Δ*gacS* background	This study
Δ*rsmEgacS*	*rsmE* deletion mutant in the Δ*gacS* background	This study
Δ*rsmAcsrAgacS*	*rsmA csrA* deletion mutant in the Δ*gacS* background	This study
Δ*rsmArsmEgacS*	*rsmA rsmE* deletion mutant in the Δ*gacS* background	This study
Δ*csrArsmEgacS*	*csrA rsmE* deletion mutant in the Δ*gacS* background	This study
Δ*rsmAcsrArsmEgacS*	*rsmA csrA rsmE* deletion mutant in the Δ*gacS* background	This study

Rif^r^: Rifampin resistance; Km^r^: Kanamycin resistance.

### Bacterial mutagenesis

Site-directed mutagenesis of the two small RNAs and three repressor protein genes was performed with the pEX18Tc suicide vector as described by de Bruijn and colleagues (de Bruijn *et al*., 2008). The primers used are listed in Table S7. For each mutant construct, two fragments were amplified: Up and down fragments. In the first-round polymerase chain reaction (PCR), the up and down fragments were amplified respectively. The first round PCR was performed with Pfu polymerase (Promega). The program used for the PCR consisting 1 min denaturation at 95°C, followed by 30 cycles of 95°C 1 min, Tm 30 s and 72°C 2 min. The last step of the PCR was 72°C for 7 min. All fragments were separated on a 1% (wt/vol) agarose gel and purified with an Illustra GFX PCR DNA and Gel Band Purification Kit. The second round PCR was performed by mixing equimolar amounts of the up and down fragments as templates, up forward and down reverse primers were added in the Pfu PCR reaction system. All fragments were separated on a 1% agarose gel, and bands of the right size were purified with a Qiagen kit. The fragments were digested with EcoRI and HindIII and cloned into pEX18Tc. *Escherichia coli* DH5α was transformed with pEX18TC-*rsmY*, pEX18TC-*rsmZ*, pEX18TC-*rsmA*, pEX18TC-*csrA* or pEX18TC-*rsmE* plasmids by heat shock transformation according to method of Inoue and colleagues (Inoue *et al*., 1990), and transformed colonies were selected on LB supplemented with 25 μg ml^−1^ tetracycline (Sigma). Integration of the inserts was verified by restriction analysis of the plasmids. The plasmid inserts were verified by sequencing (Macrogen, Amsterdam, the Netherlands). The correct pEX18Tc-*rsmY* and pEX18Tc-*rsmZ* constructs were subsequently electroporated into *P. fluorescens* SS101; pEX18Tc-*rsmA*, pEX18Tc-*csrA* and pEX18Tc-*rsmE* constructs were transformed into the Δ*gacS* mutant. Electrocompetent cells were obtained according to the method of Choi and colleagues (2006), and electroporation occurred at 2.4 kV and 200 μF. After incubation in SOC medium [2% Bacto tryptone (Difco), 0.5% Bacto yeast extract (Difco), 10 mM NaCl, 2.5 mM KCl, 10 mM MgCl_2_, 10 mM MgSO_4_, 20 mM glucose (pH 7)] for 2 h at 25°C, the cells were plated on KB supplemented with tetracycline (25 μg ml^−1^) and rifampin (50 μg ml^−1^). The single crossover colonies obtained were grown in LB overnight at 25°C and plated on LB supplemented 5% sucrose to accomplish the double crossover. The plates were incubated at 25°C for at least 48 h, and colonies were re-streaked on LB supplemented with tetracycline (25 μg ml^−1^) and on LB supplemented with 5% sucrose. Colonies that grew on LB with sucrose, but not on LB with tetracycline, were selected and subjected to colony PCR to confirm the deletion of the genes.

### Lipopeptide extraction and RP-HPLC separation

Massetolide extractions and RP-HPLC analysis were conducted according to the methods described previously (de Bruijn *et al*., 2008; de Bruijn and Raaijmakers, 2009a). Briefly, *Pseudomonas* strains were grown on Pseudomonas agar plates (Pseudomonas agar 38 g l^−1^, glycerol 10 g l^−1^) for 48 h at 25°C. The cells were suspended in sterile de-mineralized water (∼ 40 ml per plate), transferred to 50 ml tubes, shaken vigorously for 2 min and then centrifuged (30 min, 6000 rpm, 4°C). The culture supernatant was transferred to a new tube and acidified to pH 2.0 with 9% HCl. The precipitate was obtained by centrifugation (30 min, 6000 rpm, 4°C) and washed three times with acidified dH_2_O (pH 2.0). The precipitate was re-suspended in 5 ml dH_2_O and the pH adjusted to 8.0 with 0.2 M NaOH; the precipitate dissolves. The solution was centrifuged (30 min, 6000 rpm, 4°C) and the supernatant transferred to a new tube and subjected to lyophilization. Analytical HPLC separations were carried out on 5 μm C18 column (Waters Symmetry column, Waters, Etten-Leur, Netherlands), a 55 min linear gradient of 0% to 100% acetonitrile + 0.1% (v/v) trifluoroacetic acid with a flow rate of 0.5 ml min^−1^. Detection was performed with a photodiode array detector (Waters) at wavelengths from 200 to 450 nm.

### Swarming motility

Swarming motility assays of the bacterial strains and mutants were conducted according to the method described previously (de Bruijn and Raaijmakers, 2009a). Swarming motility of wild type strain SS101 and the mutants was assessed on soft (0.6% wt/vol) standard succinate agar medium (SSM) consisting of 32.8 mM K_2_HPO_4_, 22 mM KH_2_PO_4_, 7.6 mM (NH_4_)_2_SO_4_, 0.8 mM MgSO_4_ and 34 mM succinic acid and adjusted to pH 7 with NaOH. After autoclaving, the medium was cooled down in a water bath to 55°C and kept at 55°C for 1 h. Twenty millilitres of SSM was pipetted into a 9 cm diameter petri dish, and the plates were kept for 24 h at room temperature (20°C) prior to the swarming assay. For all swarming assays, the same conditions (agar temperature and volume, time period of storage of the poured plates) were kept constant to maximize reproducibility. Overnight cultures of wild-type SS101, mutants, were washed three times with 0.9% NaCl, and 5 μl of the washed cell suspension (1 × 10^10^ cells ml^−1^) was spot inoculated in the centre of the soft SSM agar plate and incubated for 48–72 h at 25°C.

### Transcriptional profiling

Wild-type SS101,the Δ*gacA* and the Δ*rsmYZ* mutant were grown in King's medium B in 24-well plates, and harvested for RNA isolation at the mid-exponential growth stage (OD600 = 0.6). Cells of these strains were collected in triplicates. Total RNA was extracted with Trizol reagent (Invitrogen) and further purified with the NucleoSpin RNA kit (Macherey-Nagel). A tiling microarray for *P. fluorescens* SS101 was developed in the MicroArray Department (MAD), University of Amsterdam (UvA), Amsterdam, the Netherlands. In total, 134 276 probes (60 mer) were designed with, in general, a gap of 32 nucleotides between adjacent probes on the same strand and an overlap by 14 nucleotides when regarding both strands. In addition, 5000 custom negative control probes were hybridized, and used as an internal control to validate the designed probes in a comparative genomic hybridization experiment of four arrays. Probes were annotated and assembled into probe sets for known genes based on location information retrieved from the Pathosystems Resource Integration Center (http://patricbrc.org). Probes outside of known genes were labelled as InterGenic Region. Complementary DNA (cDNA) labelling was conducted as described previously (52). Briefly, cDNA was synthesized in presence of Cy3-dUTP (Cy3) for the test samples and with Cy5-dUTP (Cy5) for the common reference. The common reference was made by an equimolar pool of the test samples (3 μg per sample). Five micrograms of total RNA per reaction was used and yielded 1.5–2.5 μg cDNA for each sample with more than 16 pmol of Cy3 or Cy5 dye per microgram. Hybridizations were performed according to Pennings and colleagues (Pennings *et al*., 2011). Slides were washed according to the procedures described in the Nimblegen Arrays User's Guide – Gene Expression Arrays Version 5.0 and scanned in an ozone-free room with a Agilent DNA microarray scanner G2565CA (Agilent Technologies). Feature extraction was performed with NimbleScan v2.5 (Roche Nimblegen). Data pre-processing consisted of log_2_-transformation of the raw probe-intensity data, followed by a within slide Lowess normalization. Thus, normalized sample (Cy3) channel intensities were summarized into probe sets values and normalized between arrays using the Robust Multi-Array Analysis algorithm (Irizarry, *et al*. 2003). All results described were found to be significant using a false discovery rate of less than 5%. The Arraystar 12 software (DNASTAR, Madison, Wisconsin, USA) was used for analysing the pre-normalized array data. Statistical analyses were carried out with the normalized data using a moderated *t*-test to determine differential transcript abundance. Genes with a fold change > 2 and *P*-value < 0.05 were considered to be differentially regulated.

## Supporting Information

**Fig. S1.** Phylogenetic analyses of RsmA/CsrA-like proteins in different *Pseudomonas* species and strains. The phylogenetic tree is based on amino acid sequences of RsmA, RsmE and CsrA from 23 bacterial genomes, and was generated by neighbor-joining (NJ) (Saitou and Nei, 1987) in MEGA 6 (Tamura *et al*., 2013). The evolutionary distances were computed using Jones–Taylor–Thornton (JTT) model. The variation rate among sites was modelled with a gamma distribution. Bootstrap values (1000 repetitions) are shown on branches. Rsm proteins from *P. fluorescens* strain SS101 are indicated in bold.

**Table S1.** tRNA and rRNA in SS101. **Table S2.** Whole genome transcriptome analysis of ΔgacS/wt. **Table S3.** Whole genome transcriptome analysis of ΔgacA/wt. **Table S4.** Whole genome transcriptome analysis of ΔrsmYZ/wt, up-regulated genes with *P* < 0.001, fold change > 2. **Table S5.** Whole genome transcriptome analysis of ΔrsmYZ/wt, down-regulated genes with *P* < 0.001, fold change > 2. **Table S6.** Unique expression genes in ΔgacA and ΔrsmYZ mutants. **Table S7.** Primers used for in frame deletion mutagenesis of the small RNAs and repressor proteins.
